# Association between PFO and neurological decompression illness

**DOI:** 10.1186/1824-7288-35-1

**Published:** 2009-02-07

**Authors:** Diletta de Benedictis, Francesco Marcucci

**Affiliations:** 1 Clinica Pediatrica, Università di Perugia, Perugia, Italia

Sir,

Greco et al. described a case of a 12-year-old girl who presented symptoms of stroke after diving into the sea [1]. An ischemic lesion in the lenticular nucleus, in the posterior limb of internal capsula and in the caudate nucleus of the right hemisphere was found at a Diffusion Weighted Imaging. No abnormality of coagulation parameters was revealed. Surprisingly, there is no mention of investigation of congenital heart disease, which is recognised as a major cause of stroke in children [2].

Persistence of patent foramen ovale (PFO) is present in up to 25% of general population. Under normal circumstances, this is of little relevance as the left atrial pressure is higher than that of the right and the PFO is functionally closed most of the time. However, during coughing, sneezing, diving, and other conditions of increased endothoracic pressure, the PFO can open allowing potential for right-to-left shunt. This opens the cerebral circulation to the systemic venous circulation and has been implicated in the pathogenesis of stroke [3]. In adults, the presence of PFO is associated with cryptogenic stroke [4]. It is not known whether children with such condition have a similar increase in PFO, but a causal link has been suggested [5].

SCUBA diving is popular between adolescents. There is an association between PFO and neurological decompression illness, the onset of which is soon after surfacing. Divers with this type of decompression illness have a much higher prevalence of significant right-to-left shunt than do controls [6]. An increased prevalence of brain lesions has also been reported in divers, with a right-to-left shunt present in those with multiple brain lesions [7].

Although rare, cryptogenic stroke in childhood is devastating, and investigation of PFO-associated right-to-left shunt is largely warranted in such condition [8].
